# Assessment of vaccination competency among health workers in health facilities in East Wollega, Oromia, Western Ethiopia: An observational study

**DOI:** 10.1371/journal.pone.0358136

**Published:** 2026-09-15

**Authors:** Adugna Olani, Gedefa Bayisa, Merga Chala, Lami Gurmessa, Jiregna Fayera

**Affiliations:** 1 School of Nursing and Midwifery, Institute of Health Sciences, Wollega University, Nekemte, Ethiopia; 2 Department of Public Health, Institute of Health Sciences, Wollega University, Nekemte, Ethiopia; 3 School of Medicine, Institute of Health Sciences, Wollega University, Nekemte, Ethiopia; George Washington University School of Medicine and Health Sciences, UNITED STATES OF AMERICA

## Abstract

**Background:**

Vaccination competency is a critical determinant of immunization quality and safety. However, limited evidence exists on the practical skills of frontline providers in Ethiopia.

**Methods:**

A facility-based observational study assessing vaccination competency among health care providers involved in routine childhood immunization.

**Results:**

A total of 34 providers were observed while administering 10 vaccine doses each, resulting in 340 observed vaccination encounters. Competency was assessed using a structured checklist covering patient/parent education, medical and office protocols, vaccine preparation, vaccine administration, and record-keeping. A composite competency score was calculated for each provider and expressed as a percentage. Domain-specific competency scores were summarized using descriptive statistics, and providers scoring ≥80% were classified as competent. We observed 340 vaccination procedures performed by healthcare providers in 34 health facilities. Most providers were aged 30–39 years (19/34), male (22/34), held a Bachelor of Science (23/34), and had 6–10 years of work experience (20/34). Competency varied across domains, with the highest performance in vaccine administration (71%) and the lowest in record-keeping (22%). The overall mean competency score was 50%, below the predefined 80% competence threshold.

**Conclusion:**

Moderate technical skills but failure in infection control, patient communication, and documentation resulted in weak practical competency, were observed from this study. Data indicate a gap between knowledge-based practice and actual performance. To improve immunization service, it is essential to enhance supervision and mentorship, focus on vaccine information system usage, and provide regular competency-based training for EPI providers. Strengthening routine monitoring and supportive supervision is critical, along with further research to identify barriers and assess training effectiveness. Addressing these gaps is vital for enhancing the quality and coverage of immunization services.

## Introduction

Immunization is widely acknowledged as one of the most cost – effective public health measures, and it is essential in lowering the morbidity and mortality rates of diseases that can be prevented by vaccination [1]. The World Health Organization (WHO) estimates that vaccinations avert 2–3 million deaths annually [2]. But the availability of vaccines is not the only factor that affects the quality of vaccination services; healthcare providers’ ability to follow safe and efficient procedures is also a factor. This implies using the right vaccination administration methods, following infection control guidelines, and keeping thorough records [3]

Ethiopia has achieved notable progress in increasing the coverage of childhood vaccinations. The Expanded Program on Immunization (EPI), which was put into place by the Ethiopian government, has improved vaccine accessibility nationwide [4]. According to the latest WHO/UNICEF Estimates of National Immunization Coverage (WUENIC), Ethiopia achieved 84% coverage for the first dose (DTP1) and 74% coverage for the third dose (DTP3) of the diphtheria–tetanus–pertussis vaccine in 2024, indicating that routine immunization coverage remains below national and global targets [5].. But even with these advancements, there are still issues with making sure vaccinations are given in a safe and efficient manner. There have been reports of problems like poor documentation practices, inconsistent adherence to infection prevention guidelines, and insufficient training of healthcare providers [6]

In order to reduce adverse events following immunization (AEFI) and preserve public confidence in vaccination programs, infection prevention is especially important in vaccination practices. Furthermore, accurate record-keeping is crucial for tracking immunization coverage at the community and national levels as well as for tracking each person’s vaccination status. Finding coverage gaps and putting effective interventions in place become difficult without accurate records [7].

In order to pinpoint areas that need focused capacity-building and quality improvement initiatives, it is essential to evaluate the vaccination competencies of EPI providers. Research indicates that increasing provider competencies may result in better vaccination procedures and greater rates of immunization coverage [8]. Furthermore, studies suggest that continuous training and assistance for medical professionals greatly improves health outcomes in vaccination programs [9].

In conclusion, even though Ethiopia has achieved impressive strides in childhood vaccination coverage through its EPI, it is still imperative to assess and improve the skills of those who administer vaccinations. In order to address particular issues with the vaccination process, stakeholders can create customized interventions by concentrating on competency assessment. This approach will not only improve the quality of immunization services but also ensure that all children receive safe and effective vaccinations.

One of the most inexpensive public health initiatives is immunization. However, the availability of vaccines and the ability of providers to adhere to safe and efficient procedures are both factors that affect the quality of vaccination services. Although childhood vaccination coverage has increased in Ethiopia, there are still issues with safe administration, infection prevention compliance, and accurate record-keeping. Evidence for focused capacity-building and quality improvement can be obtained by evaluating vaccination competencies.

## Objectives

### General objective

- To assess the competency of health workers regarding safe vaccination practices in the immunization service areas of Western Ethiopia, October–November 2025

### Specific objectives

- To determine health workers’ practices related to patient or caregiver education and informed consent before vaccine administration.
- To assess health workers’ adherence to recommended vaccine preparation and administration techniques.
- To assess health workers’ compliance with infection prevention measures during vaccination.
- To assess health workers’ preparedness to manage AEFI, including anaphylaxis.
- To assess health workers’ practices related to vaccination documentation and use of immunization records.

## Materials and methods

### Study design and setting

This facility-based cross-sectional study was conducted in East Wollega Zone, Oromia Regional State, Ethiopia from October to November, 2025. The zone has 69 public health facilities, including hospitals and health centers, providing routine healthcare services including childhood immunization through the Ethiopian EPI. Thirty-four public health facilities providing routine vaccination services were included in the study. Facilities were selected from those providing childhood immunization services during the study period, as the study aimed to assess competencies of health workers involved in vaccine delivery.

### Study population and sampling

The study included 34 EPI providers from 34 health facilities. One EPI provider responsible for routine vaccination services on the day of data collection was included from each facility. Each provider was observed while vaccinating 10 children.

### Eligibility criteria

#### Inclusion criteria.

- Licensed health care providers (e.g., nurses, midwives, health officers) involved in routine childhood immunization services
- Were actively providing vaccinations under the EPI during the study period
- Had at least 6 months of experience in immunization service delivery at the study facility
- Were present and on duty during the data collection period
- Provided informed consent to be observed while administering vaccines

#### Exclusion criteria.

- Were students, interns, or trainees not independently responsible for vaccine administration
- Were on leave, reassigned, or not providing immunization services during the data collection period
- Performed fewer than 10 observed vaccination encounters during the observation period

### Sample size

The study included 340 vaccinations observation from 34 EPI providers. Each provider was observed while vaccinating 10 children, resulting in 34 × 10 = 340 observed child vaccination encounters.

### Vaccination services assessed

Observations were conducted during routine childhood immunization sessions. Vaccines administered included those in the Ethiopian Expanded Program on Immunization (EPI), such as Bacille Calmette–Guérin (BCG), oral polio vaccine (OPV), pentavalent, pneumococcal conjugate vaccine (PCV), rotavirus, and measles-containing vaccine (MCV), according to the national immunization schedule. Providers were observed while administering vaccines appropriate for the child’s age.

### Data collection tools and procedures

Ethical approval was granted by the Wollega University Institutional Review Board. Written informed consent was obtained from EPI providers and caregivers before data collection, and confidentiality was maintained throughout the study. Data were collected during routine childhood immunization sessions using a structured competency assessment checklist adapted from the Ethiopian National EPI guidelines and the WHO immunization standards. Vaccines administered during the observations included those recommended under the national childhood immunization schedule, such as BCG, OPV, pentavalent vaccine, PCV, MCV), according to the child’s age. One trained data collector directly observed each EPI provider while vaccinating 10 children without interfering with routine service delivery. Observable competencies, including caregiver communication, vaccine preparation, infection prevention practices, vaccine administration, and documentation, were assessed through direct observation. Facility-level practices, such as cold chain monitoring, were verified through observation of equipment and available records, while provider characteristics were confirmed through interview and supporting documentation where available.

### Data analysis

Competency was assessed using a structured observational checklist. Each item was scored as ‘1’ if performed correctly and ‘0’ if not performed. Each EPI provider was observed while vaccinating 10 children. Because observations were repeated for each provider, child-level observations were considered clustered within providers. Child-level competency indicators were summarized across the 340 observed vaccination encounters, while provider-level competency scores were calculated by aggregating observations from the 10 encounters for each provider and converting the total score into a percentage. Variability between providers was assessed by comparing the distribution and range of competency scores across the 34 providers. Analyses were conducted at both child-observation and provider levels, depending on the nature of the competency indicator. While no explicit national numeric cut-off exists, the ≥ 80% threshold is widely used in WHO-supported programs and comparable studies and was therefore adopted as the competency benchmark.

### Variables and measurements

The outcome variable was vaccination competency, assessed through overall, domain-level, and item-level performance. Independent variables included selected health worker characteristics: age, sex, educational qualification, and work experience. Descriptive statistics were used to summarize competency scores across domains and individual items.

### Ethics statement

Ethics approval was obtained from the Institute of Health Sciences Research, Technology Transfer and Postgraduate Study Directorate of Wollega University (Reference No. IHSRPTTAD111/2014). Participant recruitment and direct observation were conducted from 1 October 2025–30 November 2025. Verbal informed consent was obtained from all healthcare providers before observation. Verbal consent was documented by the data collectors on the observation checklist before commencement of each observation session. Confidentiality and anonymity of participants were maintained throughout the study.

## Results

A total of 340 vaccination procedures were directly observed across 34 facilities. Providers’ competencies were evaluated in five domains: patient/parent education, medical and office protocols, vaccine preparation, vaccine administration, and record-keeping.

### Sociodemographic and professional characteristics of study participants

The sociodemographic data reveals a diverse sample of participants across various categories. In terms of age, the majority fall within the 30–39 age group, comprising 19 individuals, while 14 participants are aged between 20 and 29. There is a notable decrease in representation for those aged 40 and above, with only one participant in the 40 + category. Gender distribution shows a predominance of males, with 22 male participants compared to 12 females. Regarding education levels, most participants hold a Bachelor of Science degree, totaling 23 individuals, followed by 8 participants with a diploma and 3 with a Master’s degree or Master of Public Health (MSc/MPH). Lastly, work experience varies among the participants; 20 individuals have between 6–10 years of experience, while 10 have 1–5 years, and only 4 participants have over 11 years of experience

### Competency assessment result

**Fig 1:**
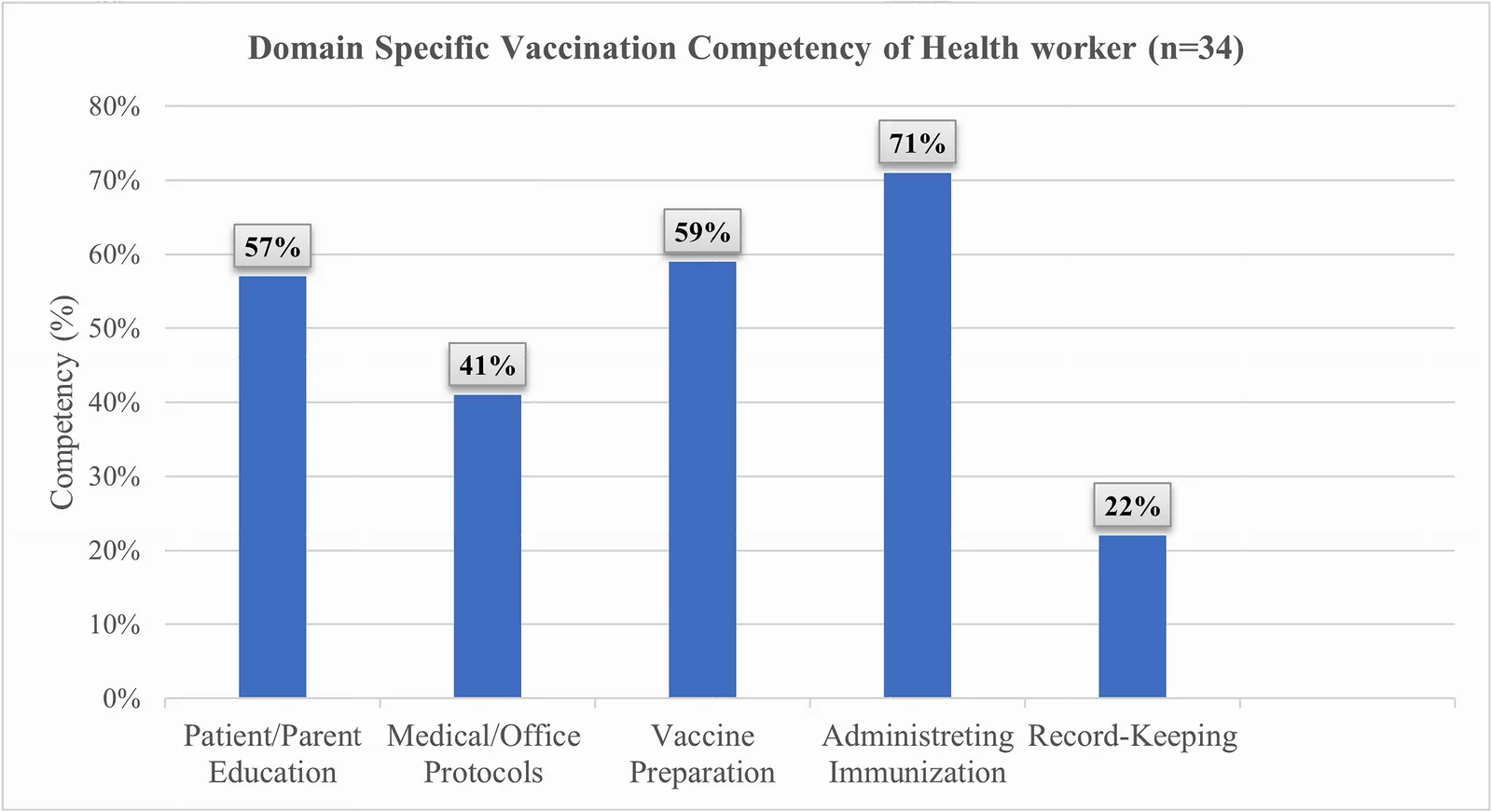
Domain-specific vaccination competency among EPI providers based on observed vaccination practices (n = 34 providers and 340 child vaccination encounters).

Domain-specific vaccination competency among healthcare workers varied across the assessed areas (Fig 1) The highest competency level was observed in administering immunizations, with 71% of healthcare workers meeting or exceeding competency criteria. Vaccine preparation was the second highest domain, with 59% meeting/exceeding competency, followed by patient/parent education (57%). Competency was lower in medical and office protocols, where 41% of healthcare workers met/exceeded the competency criteria. Record-keeping demonstrated the lowest competency level, with 22% of healthcare workers meeting/exceeding the required criteria.

Child-level vaccination practices were assessed through direct observation of immunization encounters. In the patient/parent education domain, 65% of observed encounters included welcoming the patient/family and establishing rapport. Providers explained vaccines and injection types in 68% of encounters, addressed questions and literacy or language barriers in 79%, screened for contraindications in 56%, and reviewed comfort measures and post-vaccination care in 68%.

During vaccine administration, patient identity was verified in 68% of observed encounters. Correct positioning or restraint and identification of the injection site were performed in 85% of encounters, while anatomical landmarks were correctly located in 94%. The correct vaccine route was demonstrated in 74% of encounters, needle insertion at the correct angle in 82%, and correct needle withdrawal after injection in 74%. Post-injection pressure application was performed in 94% of encounters. Strategies to reduce anxiety or pain were used in 56% of encounters, while proper sharps disposal and vial disposal were observed in 79% and 82% of encounters, respectively (Table 1).

**Table 1:** Child-level vaccination competency among observed vaccination encounters (n = 340).

Competency domain	Competency item	Meets/exceeds competency (%)
Patient/Parent Education	Welcomes patient/family and establishes rapport	65%
	Explains vaccines and type of injection	68%
	Answers questions/addresses literacy or language barriers	79%
	Screens for contraindications	56%
	Reviews comfort measures and aftercare	68%
Administering Immunizations	Verifies patient identity	68%
	Positions/restraints correctly	85%
	Identifies injection site	85%
	Locates anatomic landmarks	94%
	Demonstrates correct vaccine route	74%
	Inserts needle at correct angle	82%
	Injects and withdraws needle correctly	74%
	Applies pressure post-injection	94%
	Uses strategies to reduce anxiety/pain	56%
	Proper sharps disposal	79%
	Proper vial disposal	82%

Provider-level competency varied across the assessed vaccination practice areas. In the patient/parent education domain, only 6% of providers verified receipt of Vaccine Information Statements (VIS). For medical and office protocols, competency levels ranged from 21% for identifying the location and indication of epinephrine to 56% for demonstrating proper vaccine handling. Other competencies in this domain included identifying the location of medical protocols (44%), maintaining Cardiopulmonary Resuscitation (CPR) certification (50%), and reporting needlestick injuries and maintaining sharps logs (32%).

Within the vaccine preparation domain, competency ranged from 6% for performing hand hygiene before preparation and maintaining aseptic technique during septum cleaning to 85% for checking vial expiration dates/labels. Other observed competencies included checking refrigerator/freezer temperature (59%), preparing vaccines in a clean medication area (68%), selecting the correct needle size (79%), and preparing vaccines according to manufacturer instructions (76%). For safe injection practices, 74% of providers demonstrated adherence to recommended practices, and 74% appropriately labeled filled syringes.

Provider competency in record-keeping activities was comparatively lower. Only 18% of providers fully documented vaccination details, including lot number, injection site, VIS, and initials. Similarly, 26% used an immunization registry/computer system, and 21% updated patient vaccination record cards (Table 2)

**Table 2:** Provider level vaccination competency among EPI providers (n = 34).

Competency domain	Competency item	Meets/exceeds competency (%)
Patient/Parent Education	Verifies receipt of VIS	6%
Medical and Office Protocols	Identifies location of medical protocols	44%
	Identifies epinephrine location/indication	21%
	Maintains CPR certification	50%
	Reports needlestick injuries and maintains sharps log	32%
	Demonstrates proper vaccine handling	56%
Vaccine Preparation	Performs hand hygiene before preparation	6%
	Checks refrigerator/freezer temperature	59%
	Checks vial expiration/label	85%
	Prepares in clean medication area	68%
	Selects correct needle size	79%
	Maintains aseptic technique (septum cleaning)	6%
	Prepares vaccine per manufacturer instructions	76%
	Adherence to safe injection practices	74%
	Labels filled syringes	74%
Record-Keeping	Fully documents vaccination (lot, site, VIS, initials, etc.)	18%
	Uses immunization registry/computer system	26%
	Updates patient vaccination record card	21%

## Discussion

The present study identified substantial variation in vaccination competencies across different domains. Although providers demonstrated good performance in several technical aspects of vaccine administration, particularly injection site identification and anatomic landmark recognition, important deficiencies were observed in infection prevention, documentation, and adherence to immunization protocols. These findings suggest that targeted in-service training, supportive supervision, and regular competency assessments are needed to strengthen routine immunization practices and improve the quality and safety of vaccination services.

This study revealed significant gaps in vaccination practices. While providers showed adequate technical injection skills, infection prevention practices were alarmingly poor, echoing findings from Addis Ababa infection control studies where hand hygiene compliance was < 10%. Documentation also remained weak, consistent with national assessments of immunization data quality in Ethiopia. The extremely low use of VIS/informed consent (2%) highlights a gap in patient education, which is critical for informed consent and vaccine confidence. The strong link between education, experience, and competency suggests that continuous professional development is essential.

In this study, adherence to hand hygiene remained critically low at 5%, and aseptic technique for vial septum cleaning was only 3%. Glove use during administration was also observed at just 3%. These findings are consistent with an infection control study in Addis Ababa, Ethiopia, which reported poor compliance with hand hygiene before patient contact (~7%). While other studies often report higher compliance in self-reports, our observational findings demonstrate much lower real-world adherence, underscoring the gap between knowledge and practice [10,11].

Our study found only moderate performance in cold chain management and handling practices. For example, 60% checked refrigerator/freezer temperatures, 71% checked vial expiration/label, and 57% demonstrated correct vaccine handling. This contrasts with earlier cold chain studies in Oromia, which found ~50–60% good practice. Although some aspects (e.g., vial checking) were stronger in our study, overall cold chain adherence is still insufficient, suggesting continued challenges in consistent implementation [12]

Documentation remains the weakest domain. In our updated results, only 10% fully documented vaccinations, 25% updated patient cards, and 45% used digital registries. These results are consistent with national-level assessments of Ethiopia’s immunization program, which have highlighted poor data quality, discrepancies in reporting, and limited use of registries. Our observational data reinforces that documentation is a persistent systemic gap [6].

The updated results show that 65% of providers established rapport and 69% explained vaccines, while 80% answered questions and addressed literacy/language barriers. However, only 2% verified VIS use, and only 55% screened for contraindications. These findings mirror other reports from LMICs, where verbal communication is more common than providing standardized written materials. Our 2% VIS provision rate is particularly low compared with international standards [9]

Awareness of emergency protocols remains inadequate in the updated results. Only 30% identified epinephrine storage and use, and 50% identified medical protocols. Similar findings are echoed in infection control and safety studies in Ethiopia, which have reported low awareness and preparedness for emergency response. This underlines the need for routine drills and protocol reinforcement [13].

Overall, the updated results confirm patterns reported in previous Ethiopian and regional studies. While some technical skills such as rapport building and injection technique were moderate to strong, infection prevention, documentation, VIS provision, and emergency preparedness remain critically weak. The reliance on direct observation in this study revealed even lower actual practice levels compared to self-reported rates in other studies, highlighting the urgent need for training, supervision, and behavioral interventions to improve immunization service quality.

### Limitations and methodological transparency

Several methodological limitations should be considered when interpreting the findings. First, although the study included 34 facilities across the region, facility selection was not randomized, limiting the representativeness and generalizability of results to all health facilities in western Ethiopia.

Second, only one EPI provider was observed per facility. This may not capture intra-facility variation in performance, and provider selection was based on who was actively vaccinating on the day of the visit, introducing potential selection bias.

Third, although measures were taken to minimize the Hawthorne effect—such as reassuring providers that observations were confidential and would not be used for performance evaluation—some degree of behavior change due to being observed may still have occurred

Fourth, the clustered nature of the data reduces the effective sample size. Although 340 observations were collected, the true analytical power reflects the number of clusters (34 providers). Failure to account for clustering could underestimate standard errors and overstate statistical significance.

Fifth, although data collectors were trained, the study did not include formal assessment of inter-rater reliability (e.g., kappa statistics), which may introduce observer variability

Finally, confidentiality requirements prevented linking provider performance to specific facility characteristics. This limits exploration of contextual factors (workload, supervision, supply availability) that may influence performance outcomes.

Despite these limitations, the study provides important insights into EPI provider competencies across multiple health facilities and identifies practical areas for quality improvement in routine immunization service delivery.

## Conclusion and recommendations

### Conclusion

The vaccination providers demonstrate moderate technical skills but major weaknesses in infection prevention, patient communication, and documentation. In this study, no statistically significant correlation was found between the dependent and independent variables. However, direct observation during data collection revealed that the competency of EPI focal persons in practice was generally low. This finding highlights an important gap between theoretical knowledge and actual performance in immunization service delivery. While the absence of correlation may indicate that the selected variables are not strong predictors of competency, the observed low performance suggests that other unmeasured factors—such as training, supervision, workload, or motivation—may play a critical role.

### Recommendations

1. Ensure supervision and mentorship with emphasis on VIS use and patient education.
2. Integrate competency-based refresher courses for EPI providers.
3. Provide regular in-service training and refresher courses for EPI focal persons to strengthen practical skills.
4. Strengthen routine monitoring, mentorship, and supportive supervision at health facility level.
5. Further Research:•
  - Conduct studies with larger samples or alternative study designs to explore other factors influencing competency.
  - Employ qualitative methods (e.g., interviews, focus groups) to gain deeper insights into barriers affecting practice
  - Consider intervention-based studies to assess the effectiveness of training and supervision programs.
6. Policy Implication: The observed competency gap should be addressed at program and policy level to improve immunization service quality and coverage.

**Key messages**

**What is already known on this topic**

Vaccination programs in low- and middle-income countries rely on competent health workers to ensure safe and effective immunization services. However, health workers’ knowledge and practices can be inconsistent, leading to vaccine wastage and reduced immunization coverage. In Ethiopia, there is limited evidence on vaccinators’ competencies and operational challenges.

**What this study adds**

This study provides primary data on the knowledge, attitudes, and practices of Ethiopian vaccinators regarding cold chain management. It identifies specific gaps that may compromise vaccine effectiveness and program quality. These findings offer concrete targets for workforce training and improved operational protocols.

**How this study might affect research, practice or policy**

Results can guide targeted interventions to strengthen vaccine storage and handling in public health facilities. Policymakers can use these insights to enhance training, supervision, and monitoring systems for vaccinators. Future research can evaluate interventions aimed at improving immunization quality and coverage in Ethiopia and similar Low- and Middle-Income Countries (LMICs)

## Abbreviations

AEFI: Risk of Adverse events following immunization
BCG: Bacille Calmette–Guérin
CPR: Cardiopulmonary Resuscitation
DTP: diphtheria–tetanus–pertussis
EPI: Expanded Program on Immunization
LMIC: Low- and Middle-Income Countries
MCV: measles-containing vaccine
OPV: oral polio vaccine
PCV: pneumococcal conjugate vaccine
UNICEF: United Nations Children’s Fund
VIS: Vaccine Information Statements
WHO: World Health Organization
WUENIC: WHO/UNICEF Estimates of National Immunization Coverage
