# Supplementary material for: Assessment of vaccination competency among health workers in health facilities in East Wollega, Oromia, Western Ethiopia: An observational study
Source: PLoS One. 2026 Sep 15;21(9):e0358136. doi: 10.1371/journal.pone.0358136 (PMC13577397; doi:10.1371/journal.pone.0358136)
Supplement: Supplementary file 1 [file pone.0358136.s001.docx]

**General comments**

Health care/Vaccine providers are critical to the delivery of immunization programmes. This is a valuable study because it investigates competencies of providers within a Low- and Middle-Income setting. The study lacks detail particularly within the methodology and results section. These additional details would enhance the quality of the article and the utility of the study for the readers.

**Specific Comments**

1. **Line 61 -65**

What is already known on this topic

***Vaccination programs in low- and middle-income countries often face challenges in maintaining. vaccine potency due to gaps in cold chain management.***

I would consider excluding this statement from the abstract as this does not describe the study, which extends beyond cold-chain management. Specifically, this study did not evaluate the cold chain through objective examination of temperature monitoring and recording. Information regarding the cold chain was according to self-reported or observed competency (checking refrigerator temperatures), rather than documenting what these temperatures were including any breaches.

2. **Line 95**

***The 2016 Ethiopian Demographic and Health Survey (EDHS) found that about 83% of the country's***

Can the authors provide a more up to date assessment of vaccine coverage?

3. **Line 100**

***In order to reduce the risk of adverse events after immunization (AEFI)***

This should be Adverse events **following** immunisation

4. **Line 160-206**

The methodology section requires further detail, specifically please address these points;

a. Study design and setting – to provide a broader context.
   i. How many health facilities are there in Western Ethiopia as this will provide an indication of what percentage were sampled.
   ii. Were these sites selected only on the basis of providing immunisations?
b. Study Population and Sampling
   i. 34 EPI providers were selected – what proportion of all EPI providers at these sites does this represent?
c. Sample Size
   i. 340 vaccination sessions observed – is this 10 sessions per provider and if so this would be observation of 100 children per provider (10X10) Please clarify?
d. Data Analysis
   i. The provider was scored on each child using the same competency check list – however, some of these competencies are “session specific” and some “child specific”. Could this be analysed separately ?
   ii. Could the data be analysed in greater depth? That is for each provider was the competencies consistent across individual children (intra-observer variability) and for each competency what was the variability between providers (inter-observer variability) ?
e. Eligibility Criteria
   i. This is provider specific and should be listed under study population and sampling
f. Other
   i. Could the authors provide a description of the vaccines administered and to which age groups.
   ii. Could the authors outline if these competencies link to national recommendations and guidelines. That is, what are providers educated and taught to do against which their competencies can be matched?
   iii. Could the authors clarify which competencies are assessed using self-report and which through observation/documentation?

5. **Line 247……**

**Inclusion of discussion points in the results section**

Could the authors ensure that interpretation of results is included in the discussion section, rather in the results section (which should a documentation of the results). An example is provided below.

*These findings suggest that while certain technical competencies are being performed well, 259 significant gaps exist in crucial areas such as record-keeping and adherence to medical protocols, 260 highlighting the need for targeted training and reinforcement of best practices to enhance patient 261 care and safety. By addressing these gaps and promoting a culture of excellence in immunization 262 administration, healthcare providers can optimize outcomes, reduce errors, and ultimately enhance 263 the overall quality of care delivered to patients (Table 2).*

6. **Line 278**

***In this study, adherence to hand hygiene remained critically low at 5%, and aseptic technique for vial septum cleaning was only 3%..............***

The issue of injection technique is critical given the risk of immunisation errors with injection site infection. Could the authors elaborate on the competency “***Uses sterile syringe/needle for each injection”.*** Under this paragraph as it would seem alarming that approximately 25% of providers are NOT using sterile syringes and needles. Does this imply that the mandated use of auto-disable syringes are not used in the Ethiopian context. Although not part of this study do the authors then have any comments about the prevalence of injection site infection in the Ethiopian context?

7. **Line 325**

***Failure to account for clustering could underestimate standard errors and overstate statistical significance***

No statistical analyses were performed as this was purely a descriptive study. From the data collected any statistical associations would be difficult to evaluate. However, did the authors look at any correlation between provider work experience, age, gender and competency scores?
