# Supplementary material for: Assessment of vaccination competency among health workers in health facilities in East Wollega, Oromia, Western Ethiopia: An observational study
Source: PLoS One. 2026 Sep 15;21(9):e0358136. doi: 10.1371/journal.pone.0358136 (PMC13577397; doi:10.1371/journal.pone.0358136)
Supplement: Supplementary file 2 [file pone.0358136.s002.docx]

**General comments**

Health care/Vaccine providers are critical to the delivery of immunization programmes. This is a valuable study because it investigates competencies of providers within a Low- and Middle-Income setting. The study lacks detail particularly within the methodology and results section. These additional details would enhance the quality of the article and the utility of the study for the readers.

**Response:** We sincerely thank the reviewer for the positive assessment of the importance of this study and for the constructive suggestions to improve the manuscript. We agree that additional methodological and results-related details were needed to improve transparency, interpretation, and the usefulness of the findings for readers. In response to these comments, we have revised the manuscript accordingly.

For clarity, our point-by-point responses are provided in **blue font immediately following each reviewer comment**, and all changes made in the revised manuscript are indicated using **Track Changes/highlighted revisions**. We have expanded the Methods section to provide additional details on the study population, sampling, vaccination services assessed, competency assessment tools, alignment with national and WHO immunization guidelines, assessment procedures, and data analysis. We have also revised the Results and Discussion sections to improve clarity and ensure appropriate separation of findings from interpretation.

**Specific Comments**

1. **Line 61 -65**

What is already known on this topic

***Vaccination programs in low- and middle-income countries often face challenges in maintaining. vaccine potency due to gaps in cold chain management.***

I would consider excluding this statement from the abstract as this does not describe the study, which extends beyond cold-chain management. Specifically, this study did not evaluate the cold chain through objective examination of temperature monitoring and recording. Information regarding the cold chain was according to self-reported or observed competency (checking refrigerator temperatures), rather than documenting what these temperatures were including any breaches.

**Response:** Thank you for this important comment. We agree that the original statement regarding cold chain management was too broad and could imply that the study evaluated vaccine potency, temperature monitoring records, or cold chain breaches, which were not assessed in this study. We have removed this statement from the abstract and revised the "What is already known on this topic" section to better reflect the focus of our study, which was the assessment of health worker vaccination competency across multiple domains, including vaccine preparation, administration, communication, and record-keeping.

2. **Line 95**

***The 2016 Ethiopian Demographic and Health Survey (EDHS) found that about 83% of the country's***

Can the authors provide a more up to date assessment of vaccine coverage?

Response: Thank you for this helpful suggestion. We agree that the 2016 EDHS estimate is outdated. We have revised the manuscript to cite the most recent WHO/UNICEF Estimates of National Immunization Coverage (WUENIC), which provide updated national immunization coverage estimates for Ethiopia.

3. **Line 100**

***In order to reduce the risk of adverse events after immunization (AEFI)***

This should be Adverse events **following** immunisation

**Response:** Thank you for this correction. We have revised the terminology throughout the manuscript and replaced "adverse events after immunization (AEFI)" with the correct term "adverse events following immunization (AEFI)" in accordance with WHO terminology.

4. **Line 160-206**

The methodology section requires further detail, specifically please address these points;

a. Study design and setting – to provide a broader context.
   i. How many health facilities are there in Western Ethiopia as this will provide an indication of what percentage were sampled.

**Response:** Thank you for this suggestion. We have clarified the study setting by specifying that the study was conducted in East Wollega Zone, Oromia Region, Western Ethiopia, rather than broadly referring to Western Ethiopia. East Wollega Zone has 68 public health facilities providing healthcare services, including routine immunization. The 34 health facilities included in this study represented 50% of the public health facilities in the zone.

ii. Were these sites selected only on the basis of providing immunisations?

**Response:** Thank you for this important clarification. The health facilities were not selected solely because they provided immunization services. Facilities were selected from public health facilities that provided routine childhood vaccination services through the Ethiopian Expanded Programme on Immunization (EPI), as the study aimed to assess the competency of health workers directly involved in vaccine delivery. Therefore, availability of routine immunization services was an eligibility criterion for facility inclusion.

b. Study Population and Sampling
   i. 34 EPI providers were selected – what proportion of all EPI providers at these sites does this represent?

Response: We have clarified the selection of EPI providers in the Methods section. One EPI provider responsible for routine vaccination services on the day of data collection was selected from each participating health facility. Therefore, the 34 selected EPI providers represented the providers delivering vaccination services at the 34 included facilities during the data collection period. We have added this clarification to the revised manuscript.

c. Sample Size
   i. 340 vaccination sessions observed – is this 10 sessions per provider and if so this would be observation of 100 children per provider (10X10) Please clarify?

Response: Thank you for this comment. We have clarified the sample size description. The 340 vaccination observations represent **10 vaccination /child observations per EPI provider** (34 providers × 10 children = 340 observations). Each provider was observed while vaccinating 10 individual children; therefore, the study included 340 child–provider vaccination observations in total. The manuscript has been revised to avoid ambiguity

d. Data Analysis
   i. The provider was scored on each child using the same competency check list – however, some of these competencies are “session specific” and some “child specific”. Could this be analysed separately?

Response: Thank you for this important observation. We agree that the competency checklist included both child-specific and session/provider-level indicators. In the revised manuscript, we have clarified that child-specific competencies were assessed across the 340 observed vaccination encounters, whereas session/provider-level competencies were summarized based on the 34 EPI providers. We have revised the Data Analysis and Results sections accordingly to avoid inappropriate repetition of session-level indicators across multiple child observations.

ii. Could the data be analysed in greater depth? That is for each provider was the competencies consistent across individual children (intra-observer variability) and for each competency what was the variability between providers (inter-observer variability) ?

**Response:** Thank you for this valuable suggestion. We agree that repeated observations of each provider provide an opportunity to assess both within-provider consistency and between-provider variability. In the revised analysis, we have added provider-level assessment by calculating competency scores for each provider across the 10 observed vaccination encounters. We have also summarized the range of competency performance across providers for each domain to demonstrate variability in vaccination practices. Additionally, we clarified that observations were clustered within providers (34 providers, 10 observations per provider).

e. Eligibility Criteria
   i. This is provider specific and should be listed under study population and sampling.

**Response:** Thank you for this comment. We agree that the eligibility criteria were specific to EPI providers rather than a separate study component. We have moved the eligibility criteria to the "Study Population and Sampling" section and clarified the selection of participating providers.

f. Other
   i. Could the authors provide a description of the vaccines administered and to which age groups.

**Response:** Thank you for this helpful suggestion. We have added a new subsection, **"Vaccination Services Assessed,"** to the Methods section. This subsection describes the routine childhood vaccines included in the observations and their corresponding target age groups according to the Ethiopian National Expanded Programme on Immunization (EPI) schedule.

ii. Could the authors outline if these competencies link to national recommendations and guidelines. That is, what are providers educated and taught to do against which their competencies can be matched?

**Response:** Thank you for this important comment. We have clarified in the **"Data Collection Tools and Procedures"** section that the competency assessment checklist was adapted from the Ethiopian National Expanded Programme on Immunization (EPI) guidelines and the World Health Organization (WHO) immunization standards. The assessed competencies reflect the expected practices and performance standards for routine childhood immunization services in Ethiopia.

iii. Could the authors clarify which competencies are assessed using self-report and which through observation/documentation?

**Response:** Thank you for this valuable suggestion. We have revised the **"Data Collection Tools and Procedures"** section to specify the assessment methods used for each competency domain. Practical vaccination competencies were assessed through direct observation of routine immunization sessions, facility-related practices were verified through observation and review of available records, and provider characteristics, where applicable, were obtained through provider interview and verified using supporting documentation when available.

5. **Line 247……**

**Inclusion of discussion points in the results section**

Could the authors ensure that interpretation of results is included in the discussion section, rather in the results section (which should a documentation of the results). An example is provided below.

*These findings suggest that while certain technical competencies are being performed well, 259 significant gaps exist in crucial areas such as record-keeping and adherence to medical protocols, 260 highlighting the need for targeted training and reinforcement of best practices to enhance patient 261 care and safety. By addressing these gaps and promoting a culture of excellence in immunization 262 administration, healthcare providers can optimize outcomes, reduce errors, and ultimately enhance 263 the overall quality of care delivered to patients (Table 2).*

Response: Thank you for this helpful comment. We agree that the highlighted text contains interpretation rather than objective presentation of the findings. We have revised the manuscript by removing these interpretive statements from the Results section and incorporating them into the Discussion section. The Results section now presents only the observed competency findings, while their implications and potential explanations are discussed in the Discussion.

6. **Line 278**

***In this study, adherence to hand hygiene remained critically low at 5%, and aseptic technique for vial septum cleaning was only 3%..............***

The issue of injection technique is critical given the risk of immunisation errors with injection site infection. Could the authors elaborate on the competency “***Uses sterile syringe/needle for each injection”.*** Under this paragraph as it would seem alarming that approximately 25% of providers are NOT using sterile syringes and needles. Does this imply that the mandated use of auto-disable syringes are not used in the Ethiopian context. Although not part of this study do the authors then have any comments about the prevalence of injection site infection in the Ethiopian context?

Response: Thank you for this important observation. We agree that the original wording of the competency item could be misinterpreted as indicating syringe or needle reuse. The item was intended to assess adherence to safe injection practices, including compliance with recommended injection procedures and the use of a new sterile auto-disable syringe and needle for each vaccination, rather than reuse of injection equipment alone. We have revised the wording in the manuscript to improve clarity. Routine childhood immunization services in Ethiopia use single-use auto-disable syringes in accordance with national EPI and WHO recommendations; therefore, our findings should not be interpreted as evidence of syringe or needle reuse. We also acknowledge that our study did not assess the prevalence of injection-site infections or adverse events following immunization.

7. **Line 325**

***Failure to account for clustering could underestimate standard errors and overstate statistical significance***

No statistical analyses were performed as this was purely a descriptive study. From the data collected any statistical associations would be difficult to evaluate. However, did the authors look at any correlation between provider work experience, age, gender and competency scores?

**Response:** Thank you for this important comment. We agree that observations were clustered because each provider was observed while vaccinating 10 children. However, this study was designed as a descriptive competency assessment, and no inferential statistical analyses, hypothesis testing, or estimation of associations were performed. Therefore, no statistical significance testing was conducted that could be affected by underestimated standard errors due to clustering. We have clarified in the Data Analysis section that observations were summarized at both child-observation and provider levels.

Regarding the potential association between provider characteristics (age, gender, and work experience) and competency scores, these analyses were not performed because the study was primarily designed to describe competency levels rather than identify predictors of competency. Additionally, the sample included only 34 providers, limiting the statistical power for multivariable analysis. We have acknowledged this as a limitation in the revised manuscript.
